# Impact of low-level viremia on HIV non-viral load suppression in low and middle-income countries

**DOI:** 10.1097/MS9.0000000000003272

**Published:** 2025-04-10

**Authors:** Jackline V. Mbishi, Adrian Koola, Haji M. Ally, Biruk D. Ayalew, Rebecca M. Sileshi, Muhidin I. Hundisa, Zarin N. Rodoshi, Saw W. Htoo, Hafidha M. Bakari, Zuhura M. Ally, Hassan F. Fussi, Emilie Ludeman, Taylor Lascko, Celestine A. Buyu, Habib O. Ramadhani

**Affiliations:** a Department of Epidemiology and Biostatistics, Muhimbili University of Health and Allied Sciences, Dar es Salaam, Tanzania; b Amity Region High School, Woodbridge, Connecticut, USA; c Kilimanjaro Christian Medical Center, Kilimanjaro, Tanzania; d St. Paul’s Hospital Millennium Medical College, Addis Ababa, Ethiopia; e Ministry of Health, Addis Ababa, Ethiopia; f Mymensingh Medical College & Hospital, Mymensingh, Bangladesh; g University of Medicine 1, Yangon, Myanmar; h University of Dar es Salaam, Dar es Salaam, Tanzania; i District Hospital, Tanga, Tanzania; j District Hospital, Dar es Salaam, Tanzania; k Health Services and Human Services Library, University of Maryland Baltimore, Baltimore, Maryland; l Center for International Health, Education, and Biosecurity, University of Maryland School of Medicine, Baltimore, Maryland

**Keywords:** low level viremia, low-mid income countries, non-viral load suppression

## Abstract

**Background:**

The World Health Organization (WHO) defined low-level viremia (LLV) as a viral load (VL) of 51–999 copies/mL, and LLV has been associated with an increased risk of virological failure and drug resistance. Limited information is available from low- and mid-income countries (LMICs), which predominantly use WHO guidelines in HIV program monitoring. We estimated pooled prevalence of LLV, non-viral load suppression (VLS), and association between LLV and non-VLS among people living with HIV in LMICs.

**Materials and methods:**

In this systematic review and meta-analysis, databases were searched for articles reporting the association between LLV and non-VLS in LMICs between January 2015 and December 2023. Participants with VL ≤50 copies/mL were considered fully suppressed and those with VL ≥1000 copies/mL were non-suppressed. Using random effects models, we computed the pooled prevalence of LLV, non-VLS, and their corresponding 95% confidence intervals (CIs). We compared pooled prevalence of LLV and non-VLS between children vs adults and between studies done in Africa vs Asia.

**Results:**

Sixteen studies with 1 159 317 people living with HIV were analyzed. Overall, pooled prevalence of LLV was 19.7% (95% CI: 15.8–23.6) and that of non-VLS was 8.6% (95% CI: 6.5–10.7). Prevalence of LLV was significantly higher among children compared to adults (25.8% vs 17.2%; *P* < 0.001) and higher among studies done in Africa compared to those in Asia (22.3% vs 15.6%; *P* < 0.001). Prevalence of non-VLS was higher among studies involving children compared to adults (17.7% vs 5.6%; *P* < 0.001), but lower among studies done in Africa compared to Asia 8.3% vs 9.0%; *P* < 0.001). Overall, LLV increased the risk of non-VLS on a subsequent VL test compared to fully suppressed (RR = 2.6; 95% CI: 2.2–3.1).

**Conclusions:**

LLV was associated with an increased risk of non-VLS. Stakeholders should consider reviewing guidelines for the threshold of VLS given that LLV was consistently associated with increased risk of non-VLS across all groups.

## Introduction

Antiretroviral therapies (ART) have significantly improved the lives of people living with HIV (PLWH), reduced morbidity and mortality associated with HIV. Global estimates show that since 2000, about 3.4 million new pediatric HIV infections have been averted^[^1^]^. Overall, estimates show that in 2022, the number of new HIV infection was 38% fewer than it was in 2010^[^1^]^. Following the increased ART coverage, an estimated 16.6 million AIDS-related deaths have been averted over the last two decades with a 47% decrease in AIDS-related deaths since 2010^[^2^]^.

**HIGHLIGHTS**

- Nearly 20% and 9% of people living with HIV in low- and mid-income countries experienced low level viremia and non-virologic suppression.
- These outcomes vary by the type of population with children experiencing higher prevalence compared to adults.
- Low level viremia was associated with increased risk of non-virologic suppression across all study populations.

Monitoring the success of ART among PLWH involves serial measurement of viral load (VL), and for those not responding to treatment, HIV drug resistance testing is performed. Although recommended by the World Health Organization (WHO) to perform resistance testing among those not responding to treatment^[^3^]^, due to the costs associated with it, low- and mid-income countries (LMICs) predominantly rely on clinical assessment and VL testing when available^[^4^]^. Currently, WHO criteria define a VL of <1000 copies/mL as suppressed and ≥1000 copies/mL as non-suppressed^[^5^]^. Among persons with VL <1000 copies/mL, those falling within the range of 51–999 copies/mL are regarded as having low-level viremia (LLV) and have been associated with higher risk of non-viral load suppression (VLS)^[^6–8^]^ and drug resistance^[^9,10^]^.

Although a VL threshold of <1000 copies/mL has been used for several years as a success marker of ART treatment, emerging evidence shows that LLV increased the likelihood of subsequent non-VLS and drug resistance and requires further evaluation. Much of this evidence is from developed countries which use treatment guidelines with different thresholds of VLS^[^11,12^]^. Limited information is available from LMICs, which predominantly use WHO guidelines in HIV program monitoring. In addition, although some studies showed an increased risk of non-VLS among those with LLV compared to those fully suppressed, others showed an inverse relationship^[^13^]^. These contradictory findings prompted the need to produce pooled estimates in order to better understand the relationship between LLV and non-VLS.

We recognize challenges with ART treatment between children and adults. For example, young children challenges included poor palatability of medications formulations, complexities of drug administration (such as measuring, crushing, mixing), type of caregiver, and storage^[^14^]^. The predominant concerns for adolescents in HIV care are stigma, retention in care and forgetfulness^[^15^]^. Stigma, alcoholism, and forgetfulness also impact adults in HIV care^[^16^]^. These challenges collectively affect adherence to ART, a key component in achieving VLS. In addition, geographic differences exist between Asia and Africa with respect to HIV care. For example, by 2023, there were 6.7 million PLWH in Asia compared to 25.9 million in eastern, western, central, and southern Africa. Moreover, ART coverage in Asia is lower than in Africa^[^17^]^. Recognizing these disparities in HIV treatment, besides determining pooled prevalence of LLV, non-VLS, and the association between LLV and the risk of subsequent non-VLS, we further stratified pooled prevalence of these outcomes by studies that involved children vs adults and those conducted in Africa vs Asia. This stratified analysis enables us to understand the magnitude of LLV and non-VLS in these sub-groups as well as determining if the association between LLV and non-VLS is modified by age and geographic location.

## Methods

### Registration

The protocol for this systematic review has been registered in the International Prospective Registry of Systematic Review with registration number CRD42023494552^[^18^]^.

### Ethical approval

Because this was a systematic review of published manuscripts, ethical approval was not sought.

### Search strategy

Database searches were performed by the librarian (EL) in PubMed, Cochrane CENTRAL, Embase, and clinicaltrials.gov for articles published between January 2015 and December 2023. Search terms were used to capture information on low level viremia and non-VLS among PLWH in LMICs. A full search strategy can be found in supplemental materials (Supplemental Digital Content Table 1, available at: http://links.lww.com/MS9/A793). The search was restricted to papers written in English. Search results were uploaded to Covidence Systematic Review Software (Melbourne, Australia) for deduplication and screening.

### Eligibility criteria

The eligibility criteria were structured using the PICOS (i.e. population, intervention, comparison, outcome, and study design) framework^[^19^]^ as follows: PLWH as the population. Those with low level viremia as exposed/intervention groups and those who were fully suppressed as comparison group. Stratified analysis was conducted to understand differences/similarity of outcome between pooled estimates resulting from studies that involved children vs adults and those which were conducted in Africa vs Asia. Type of study population and geographic location in the stratified analyses can further be regarded as the intervention/exposed and comparison groups; non-VLS as outcome; and observational studies as study design.

### Selection criteria

**Figure 1.**
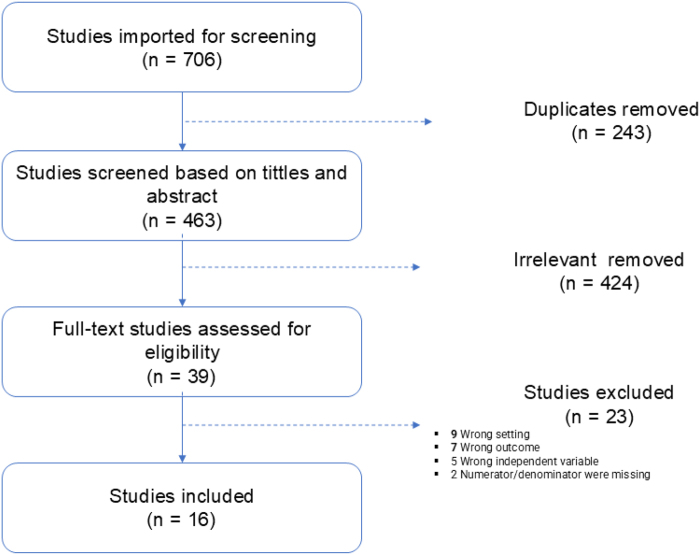
PRISMA flow diagram of the included studies for meta-analysis of impact of low-level viremia on HIV non-viral load suppression in low and middle-income countries.

Observational studies that involved PLWH in LMIC, reported association between LLV and VLS and written in English were eligible for inclusion. We excluded studies that did not report the association between LLV and VLS. A Preferred Reporting Items for Systematic Reviews and Meta-Analyses (Fig. 1) describing the literature search process and included studies is presented below. This systematic review and meta-analysis was reported according to the Preferred Reporting Items for Systematic Reviews and Meta-Analyses guidelines^[^20^]^.

### Study selection

The manuscripts searched from outlined databases were managed by Covidence software from which the final list of manuscripts was deduplicated. Two pairs of review authors (A.K./H.O.R., and R.M.S./B.D.A.) independently completed the study selection for inclusion in the appraisal process. Disagreement between two independent pairs of reviewers for the inclusion of the manuscripts was handled by the third pair of reviewers (Z.M.A./M.I.H.).

### Data extraction

Using a pre-specified Excel spreadsheet template, the two pairs of review authors (A.K./H.O.R., and R.M.S/B.D.A.) independently extracted the following data elements from the included studies: authors, year of publication, the country in which the study was conducted, study design, study period, study population (children, adults), number of PLWH, number of people with LLV, number of people with non-suppressed VL. The data were then compared, and any disagreements between the two pairs of reviewers were resolved by consensus; the third pair of reviewers (Z.M.A./M.I.H.) was consulted when necessary.

### Quality assessment

We used the National Institute for Health (NIH) tool^[^21^]^ which was designed for non-randomized studies to assess the quality of the studies because all studies included were observational. Two pairs of review authors (A.K./H.O.R., and R.M.S./B.D.A.) independently performed and rated the quality of the studies using the NIH tool. The items assessed using this tool for all studies that met inclusion criteria included reliability and validity of the measurement tools, participation rate, source of study participant recruitment, justification of sample size or power calculations, follow-up time, timing of exposure and outcome, potential confounders, and appropriateness of statistical analysis. Finally, percentage scores reflecting the quality of the studies were computed based on the stated parameters. Based on the tool, three responses (yes, no, not applicable) could be ascertained. For example, if the tool used to assess study participants was reliable, sample size was justified or power calculation was done, the item scores a “Yes” or else the item scores a “No.” A score of 1 was assigned to a “Yes” response and 0 to a “No” response. Total score was summed up and finally, percentage scores reflecting the quality of the studies were computed based on the stated parameters. A percentage score of 0–25 was considered low, 26–50 as fair, 51–75 as medium, and >75 as high quality.

### Definition of variables

#### Exposure and outcome

Exposure and outcome of interest were determined by the initial and subsequent VL results respectively. The main outcome of interest was the binary variable on the status of the VL categorized as suppressed for people who had a VL <1000 copies/mL or not suppressed for those who had a VL ≥1000 copies/mL. The main exposure of interest was a binary variable indicating LLV for those patients who had VL between 51 and 999 copies/mL and for those with a VL of ≤50 copies/mL were considered as fully suppressed.

#### Statistical analysis

Meta-regression for proportions using standard random effects models was used to compute pooled prevalence of LLV and non-VLS. The variance of proportions was stabilized using Freeman-Tukey double arcsine transformation before pooling^[^22,23^]^. To compare the effect of LLV on non-VLS, pooled risk ratio (RR) and corresponding 95% confidence intervals (CIs) were computed, again using random effects model. Subgroup analysis on the effect of LLV on non-VLS was performed based on the type of study population (children vs adults) and geographic location (Africa vs Asia). Chi-square tests were used to compare pooled prevalences of LLV and non-VLS in the stratified analysis. In both the pooled percentages and comparative analyses, we evaluated heterogeneity across studies using the *I*^2^ statistic. The *I*^2^ statistic was categorized at values of 25%, 50%, and 75% to represent low, moderate, and high extent of heterogeneity respectively as previously described^[^24^]^. The publication bias was assessed using the Egger regression asymmetry test. For both heterogeneity and publication test, a *P*-value <0.05 indicated the presence of heterogeneity and publication bias respectively. To explore the source of heterogeneity, an influential analysis using the leave-one-out method was performed. Studies with missing information, such as those that reported proportion of LLV or VLS without actual numerators and/or denominators, were excluded from the analysis. All statistical tests were performed using STATA version 17 (Stata Corporation, College Station, Texas, USA).

## Results

### Study selection

A total of 706 articles were retrieved through searches, 243 duplicates were removed. Four hundred sixty-three articles have their titles and abstracts screened and 424 were excluded. The remaining 39 received full review and 16 were determined to be eligible for final analysis (Fig. 1).

### Characteristics of studies included

Study designs from the articles included cohort studies (*n* = 16)^[^6,8,25–38^]^. Sample size for studies ranged from 172 to 597 846 people. A total of 1 159 317 PLWH were included in this analysis. We included studies from Africa (*n* = 9), and Asia (*n* = 8) (Table 1). Quality assessment showed that, all studies included were of high quality with median scores of 88% (interquartile range [83–91]).

**Table 1:** Summary of characteristics of the included studies

Author	Study period	Country	Continent	Population	Sample size	# with LLV	Quality scores %
Robinson *et al*^[^25^]^	2015–2018	Kenya	Africa	Children	172	22	91
McKenzie *et al*^[^26^]^	2021	Tanzania	Africa	Children	670	318	91
Dinesha *et al*^[^27^]^	2013–2018	India	Asia	Both	3498	533	83
Aoko *et al*^[^6^]^	2015–2021	Kenya	Africa	Both	597 846	127 015	92
Nanyeenya *et al*^[^28^]^	2016–2020	Uganda	Africa	Both	17 783	1466	85
Mnzava *et al*^[^29^]^	2017–2020	Tanzania	Africa	Adults	4177	371	85
Chun *et al*^[^30^]^	2016–2021	Nigeria	Africa	Adults	402 668	64 480	83
Sudjaritruk *et al*^[^31^]^	2008–2016	Asian countries^a^	Asia	Children	508	86	83
An *et al*^[^32^]^	2004–2018	China	Asia	Adults	93 944	21 203	92
Hsu *et al*^[^33^]^	2018	China	Asia	Adults	1078	86	91
Nzivo *et al*^[^8^]^	2015–2021	Kenya	Africa	Adults	398	280	91
Onyuro Oketch DO *et al*^[^34^]^	2005–2018	Kenya	Africa	Adults	738	165	91
Mesic *et al*^[^35^]^	2001–2017	Myanmar	Asia	Both	25 200	9861	85
Li *et al*^[^36^]^	2005–2018	China	Asia	Adults	8098	102	92
Lao *et al*^[^37^]^	2003–2023	China	Asia	Adults	830	53	83
Bareng *et al*^[^38^]^	2013–2018	Botswana	Africa	Adults	1709	43	83

LLV, low-level viremia.

^a^ Cambodia, India, Indonesia, Malaysia, Thailand, Vietnam.

### Prevalence of LLV and non-VLS

**Figure 2.**
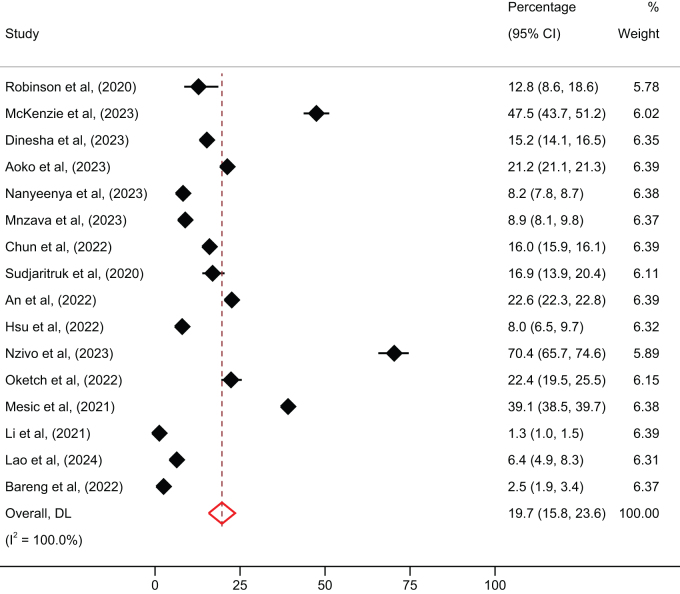
Overall prevalence of low-level viremia among people living with HIV in low- and mid-income countries.

**Figure 3.**
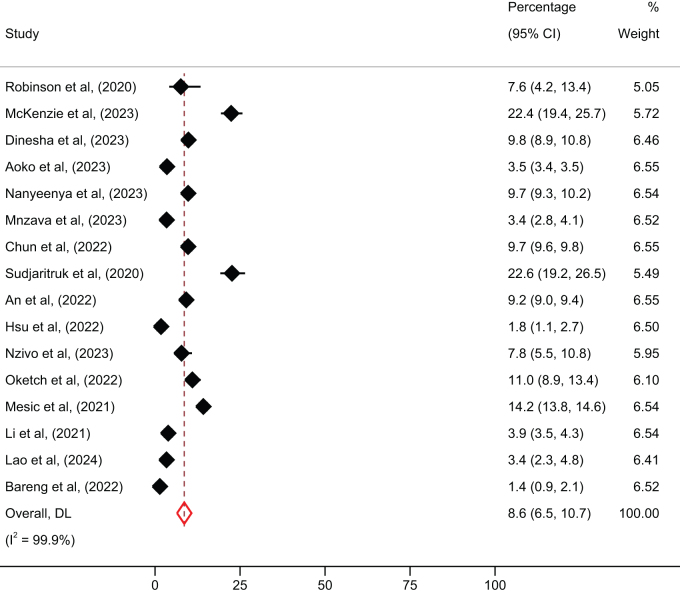
Overall prevalence of non-viral load suppression among people living with HIV in low- and mid-income countries.

Overall, pooled prevalence of LLV and non-VLS were 19.7% (95% CI: 15.8%–23.6%) and 8.6% (95% CI: 6.5%–10.7%), respectively (Figs. 2 and 3). Table 2 describes stratified analysis of the prevalence of LLV and non-VLS. There was a statistically significant difference in the prevalence of LLV among studies involving children compared to adults (25.8% vs 17.2%; *P* < 0.001). The prevalence of LLV was significantly higher among studies done in Africa compared to Asia (22.3% vs 15.6%; *P* < 0.001). Prevalence of non-VLS was significantly higher among children compared to adults (17.7% vs 5.6%; *P* < 0.001) and lower among studies done in Africa compared to Asia (8.3% vs 9.0%; *P* < 0.001).

**Table 2:** Prevalence of non-viral load suppression and low-level viremia among people living with HIV in low- and mid-income countries

Characteristic	# of studies	*n*	Prevalence (95% CI)	*I*^2^	*P*-value	Egger’s test (*z*)	*P*-value
Low-level viremia							
Overall	16	1 159 317	19.7 (15.8–23.6)	100%	0.0	2.12	0.034
Study population							
Children	3	1350	25.8 (4.2–47.3)	100%	0.0	2.34	0.019
Adults	9	513 640	17.2 (11.3–23.2)	100%	0.0	5.64	0.000
Both children and adults	4	644 327	21.0 (10.8–31.2)	100%	0.0	4.03	0.000
Geographic location							
Africa	9	1 026 161	22.3 (18.9–25.8)	100%	0.0	2.61	0.015
Asia	6	133 156	15.6 (3.7–27.8)	100%	0.0	4.17	0.000
Non-viral load suppression							
Overall	16	1 159 317	8.6 (6.5–10.7)	99.9%	0.0	2.29	0.022
Study population							
Children	3	1350	17.7 (8.9–26.4)	99.6%	0.0	3.97	0.000
Adults	9	513 640	5.6 (3.5–7.8)	99.6%	0.0	5.20	0.000
Both children and adults	4	644 327	9.3 (3.4–15.2)	99.9%	0.0	3.07	0.002
Geographic location							
Africa	9	1 026 161	8.3 (5.5–11.2)	99.6%	0.0	3.92	0.000
Asia	6	133 156	9.0 (6.5–12.3)	99.6%	0.0	2.45	0.001

CI, confidence intervals; *n*, number.

### Association between LLV and VLS

There was a higher risk of non-VLS among participants who had LLV compared to those who were fully suppressed (RR = 2.6; 95% CI: 2.2–3.1; *p* <0.001) (Supplemental Digital Content Figure 1, available at: http://links.lww.com/MS9/A789). Sub-group analysis involving children, adults, and studies done in Africa and Asia, LLV continued to be associated with an increased risk of non-VLS (Table 3).

**Table 3:** Association between low level viremia and non-viral load suppression among people living with HIV in low- and mid-income countries

Characteristics	Type of study population				Region			
	Children		Adults		Africa		Asia	
	RR (95% CI)	*P*-value	RR (95% CI)	*P*-value	RR (95% CI)	*P*-value	RR (95% CI)	*P*-value
Fully suppressed	Ref		Ref		Ref		Ref	
Low level viremia	1.8 (1.4–2.2)	<0.01	2.4 (1.9–2.9)	<0.01	2.3 (1.8–2.9)	<0.001	2.6 (2.2–3.1)	<0.01

CI, confidence intervals; RR, risk ratio.

### Assessment of heterogeneity, publication bias, and influential analysis of LLV and non-VLS

The test of heterogeneity with *I*^2^ statistic of 100% showed a significant difference (*P* < 0.001) of LLV across studies. Egger’s test (*z* = 2.12, *P*= 0.034) showed the presence of publication bias on LLV (Table 2). Leave-one-out analysis did not show a significant influence of some studies on the overall prevalence of LLV (Supplemental Digital Content Figure 2, available at:http://links.lww.com/MS9/A790). There was a significant heterogeneity of non-VLS across studies, (*I*^2^ statistic 99.9%, *P* < 0.001). Although Egger test statistic of *z* = − 2.29 (*P* = 0.022) (Table 2) showed the presence of publication bias, leave-one-out forest plot did not show a significant influence of a single study on the overall prevalence of non-VLS (Supplemental Digital Content Figure 3, available at: http://links.lww.com/MS9/A791). Regarding the association between LLV and non-VLS, there was no significant influence of a single study (Supplemental Digital Content Figure 4, available at: http://links.lww.com/MS9/A792).

## Discussion

We conducted a systematic review and meta-analysis among PLWH in LMICs. In this review, the overall prevalence of LLV was 19.7% and that of non-VLS was 8.6%. The prevalence of non-VLS was significantly higher among children compared to adults and lower among studies done in Africa compared to those in Asia. Moreover, the prevalence of LLV was higher among children compared to adults, and higher for studies done in Africa compared to those done in Asia. Overall, compared to fully suppressed, LLV was significantly associated with increased likelihood of non-VLS.

Like non-VLS, LLV has also been associated with suboptimal adherence to ART^[^39,40^]^. Overall, adherence to ART is the predominant concern in achieving VLS. To optimize the benefits of ART, interventions geared to improving medication adherence are critical. Such interventions may include enhanced adherence counseling which have proven benefits in improving adherence and achieving undetectable VL for PLWH in LMICs who had persistent viremia^[^41–44^]^. Previous reports indicated that in addition to increased risk of non-VLS and drug resistance, LLV if persistent, is associated with residual immune activation and inflammation leading to the development of non-AIDS defining events such as cardiovascular diseases and cancers^[^45,46^]^. Eventually, persistent LLV may influence morbidity and mortality associated with non-AIDS defining events^[^47,48^]^. These observations underscore the need to achieve and maintain undetectable levels of virus in order to prevent these adverse events mediated by LLV.

The findings that the overall prevalence of non-VLS was higher among children compared to adults is consistent with other studies that showed children are lagging on multiple outcomes including adherence to ART and retention in care^[^49,50^]^. Several factors described earlier negatively impact treatment outcomes among children including but not limited to HIV status nondisclosure, forgetfulness, parents/caretaker challenges including financial constraints, pill burden, and drug toxicities^[^51–53^]^. Although advanced technology has improved drug formulations, developed fixed dose combinations (to reduce pill burden and frequency of drug administration) and reduced drug toxicities in new ART medications^[^54^]^, there still exist challenges impairing children from taking their medications as prescribed. Multicomponent strategic interventions addressing HIV status disclosure, stigma, caregiver education, social support, and mental health are critical elements to improve treatment outcomes among children and adolescents. Interventions addressing these barriers have shown promising results in improving treatment outcomes among children and adolescents in LMICs^[^55–57^]^.

A few study limitations worth mentioning while interpreting these results. First, there were only three studies that involved children and adolescents compared to nine studies that involved adults. The smaller sample size for studies that involved children may not provide a better estimate of these outcomes. While there are some controversies whether LLV between 50 and 200 copies/mL is associated with an increased risk of non-VLS compared to fully suppressed^[^58–60^]^, we have not been able to provide pooled estimate by categories of LLV because different studies used different categories. Moreover, there was high degree of heterogeneity in the reported prevalences of LLV and non-virologic suppression across studies. These variations could be attributed to multiple reasons including but not limited to type of target populations, measurement instruments, timing of outcome measurements, and participants duration of treatment. We used random effect models meta-regression which assumes heterogeneous distribution as opposed to fixed effects models that assumes homogenous distribution^[^61^]^. Despite the presence of heterogeneous distribution of these outcome, no single study that had significant influence on the overall prevalence of LLV and non-virologic suppression. Furthermore, our search was limited to publications written in English; this might have excluded other potential studies not written in English. The main strength of this research is the inclusion of many studies, leading to a large sample size that enabled us to compute pooled estimates. This study remains relevant as it provides estimates of LLV and how it is associated with the risk of subsequent non-VLS among PLWH in LMIC.

## Conclusion

LLV was associated with an increased risk of non-VLS. The need to achieve and maintain undetectable levels of virus needs to be emphasized in order to minimize the risk of HIV transmission and prevent the development of non-AIDS events among patients with LLV. According to literature, both LLV and non-VLS were predominantly linked with suboptimal adherence to ART. Concerns remain in mitigating mal-adherence to optimize the benefits of new antiretroviral drugs such as dolutegravir which is the preferred first-line treatment globally^[^62^]^. As in high income countries, a more stringent threshold of VLS should be considered to monitor success of ART programs in LMICs. Such adaptations would perhaps facilitate efforts to reduce the burden of LLV and its negative consequences.

## Data Availability

All data relevant to the study are included in the article or uploaded as supplementary information. Data used for all analyses; analytic code can be requested from the corresponding author.

## Research registration unique identifying number (UIN)

PROSPERO registration number: CRD42023494552.

## Presentations of preliminary data

Preliminary data were presented at the International Conference on HIV Treatment, Pathogenesis, and Prevention Research in Resource-Limited Settings. INTEREST, Cotonou, Benin|14–17 May 2024.
